# miR‐640 Enhances Abemaciclib Sensitivity by Suppressing CDK Inhibitor Resistance‐Associated Genes in Breast Cancer Cells

**DOI:** 10.1111/jcmm.71298

**Published:** 2026-08-10

**Authors:** Ismail Mert Alkac, Cigir Biray Avci

**Affiliations:** ^1^ Department of Medical Biology Ege University Medical School İzmir Turkey

**Keywords:** abemaciclib, breast cancer, CDK4/6 inhibitor resistance, gene silencing, miR‐640

## Abstract

We investigated the potential of miR‐640 to downregulate CDK4/6 inhibitor resistance‐associated genes and evaluated its apoptotic, anti‐metastatic and anti‐proliferative effects, both alone and in combination with abemaciclib in breast cancer cells. miR‐640 was identified via GSE126125 dataset analysis. Targets were predicted using miRDB, TargetScan and TargetMiner, filtering for resistance‐associated genes. MTT assays determined IC50 values for abemaciclib and combinations. Functional effects were evaluated in MCF7 and MDA‐MB‐231 cells through Annexin V, cell cycle and wound healing assays. Target gene expression was quantified by RT‐qPCR. Successful transfection significantly upregulated miR‐640 (~50‐fold). The miR‐640/abemaciclib combination strongly inhibited migration, induced apoptosis and triggered cell line‐specific phase arrests (S and G2/M in MCF7; G0/G1 in MDA‐MB‐231). Gene expression analysis showed significant downregulation of a comprehensive resistance network (including *CDK4/6*, *CDKN2B*, *WNT7B*, *MAP3K1* and *AURKA*) in MCF7 cells. In MDA‐MB‐231 cells, *PDK1*, *CDK6*, *CDKN2B*, *SMAD2*, *ZFP91*, *MAP3K1* and *AURKA* were significantly suppressed. This study identifies miR‐640 as a potent tumour suppressor that resensitises breast cancer cells to CDK4/6 inhibition. By post‐transcriptionally downregulating key resistance genes, miR‐640—alone or additively with abemaciclib—suppresses proliferation and migration while inducing apoptosis, emerging as a promising combinatorial therapeutic strategy.

## 1 Introduction

Breast cancer (BC) accounts for approximately one‐third of all malignancies in women and remains one of the leading causes of cancer‐related mortality worldwide [1, 2]. The development of cyclin‐dependent kinase (CDK) inhibitors has revolutionised breast cancer treatment by specifically targeting uncontrolled cell cycle progression [3, 4]. Specifically, CDK4/6 inhibitors (palbociclib, ribociclib or abemaciclib) are now administered as standard targeted therapies for patients with HR+/HER2− BC. Abemaciclib, a highly specific third‐generation CDK4/6 inhibitor, provides significant clinical benefits with minimal cytotoxicity, leading to its routine clinical application [5, 6]. However, intrinsic or acquired genetic resistance developing during prolonged treatment complicates the therapeutic management, paving the way for recurrence and metastasis and ultimately worsening patient prognosis [7, 8]. Consequently, elucidating the mechanisms underlying CDK4/6 inhibitor resistance and developing novel counter‐strategies remain critical challenges in oncology [1, 9]. In this context, epigenetic and post‐transcriptional regulation via microRNAs (miRNAs) has emerged as a highly promising approach to overcome such resistance networks. By binding to the 3′UTR of target mRNAs, miRNA mimics can restore therapeutic sensitivity by simultaneously silencing multiple upregulated resistance genes [10, 11]. miRNA mimics can restore treatment sensitivity by simultaneously silencing multiple upregulated resistance genes [12]. Dysregulation in the expression profiles of miRNAs, which serve as master regulators of crucial cellular processes including apoptosis, proliferation and migration, is directly associated with cancer progression [13, 14].

In this context, miR‐640 has recently attracted attention as a context‐dependent regulatory miRNA involved in tumorigenesis, inflammatory responses and metastasis across various malignancies. While several studies have reported tumour‐suppressive functions of miR‐640 in breast and other solid tumours [15], oncogenic roles have also been described in certain cancer types, such as glioblastoma [16], underscoring its tissue‐specific and molecular background–dependent regulatory behaviour. The current literature demonstrates that the overexpression of miR‐640 inhibits cell proliferation and migration in both in vitro and in vivo BC models, suppressing tumorigenesis primarily by targeting the Wnt/β‐catenin signalling pathway via the downregulation of *WNT7B* [17]. Furthermore, miR‐640 has been reported to suppress endothelial cell migration during angiogenesis by post‐transcriptionally downregulating *ZFP91* expression [18].

Despite this accumulating evidence of its tumour‐suppressive functions, the regulatory capacity of miR‐640 specifically on gene networks associated with CDK inhibitor resistance has not been investigated to date. To address this gap in the literature, we first conducted an in silico analysis of transcriptomic data from the GEO database (GSE126125), which revealed that miR‐640 expression is significantly downregulated in relapsed breast cancer patients. Building upon this relapse‐associated finding, we hypothesised that miR‐640 could serve as a promising therapeutic agent to overcome abemaciclib resistance. To test this hypothesis, we established a comprehensive target gene panel directly or indirectly associated with CDK4/6 inhibitor resistance in the literature. Subsequently, the post‐transcriptional regulatory capacity of miR‐640 on this network, along with the in vitro functional impacts (apoptosis, cell cycle arrest and migration inhibition) of its combination with abemaciclib, were comprehensively investigated.

## 2 Materials and Methods

### 2.1 Microarray‐Identified microRNA Analysis

The GSE126125 dataset from the Gene Expression Omnibus (GEO) (https://www.ncbi.nlm.nih.gov/geo/query/acc.cgi?acc=GSE126125) was analysed using the GEO2R tool. Primary breast tumours from patients with and without relapse (*n* = 52) were evaluated. The in silico analysis revealed that miR‐640 was significantly downregulated in the relapse group (logFC: −0.4628, *p*adj: 0.0000253).

### 2.2 Target Genes Analysis

To identify potential targets of miR‐640, predicted target genes were retrieved from databases including TargetScan, miRDB and TargetMiner. Subsequently, an extensive literature review was conducted to identify genes contributing to CDKi resistance in breast cancer. These resistance‐associated genes were cross‐referenced and filtered against the miR‐640 target gene list. To visualise the functional interactions and biological significance of the selected CDKi resistance‐associated genes, a protein–protein interaction (PPI) network was constructed, and KEGG pathway enrichment analysis was performed using the STRING database (version 12.0; https://string‐db.org/). The minimum required interaction score was set to medium (0.400), and disconnected nodes were excluded to focus strictly on the highly interactive core network. Significantly enriched KEGG pathways (False Discovery Rate < 0.05) were mapped onto the network to highlight the primary biological processes regulated by these targets.

### 2.3 Cell Culture

The human breast cancer cell lines MCF7 (hormone receptor‐positive) and MDA‐MB‐231 (triple‐negative) were utilised in this study. Cells were maintained in Dulbecco's Modified Eagle Medium (DMEM, ATCC No. 30‐2002) supplemented with 10% fetal bovine serum (FBS) and 1% penicillin–streptomycin (10,000 U/mL) in a humidified incubator at 37°C with 5% CO_2_.

Cell cultures were routinely monitored for any signs of contamination and morphological integrity using inverted phase‐contrast microscopy. To ensure genetic and phenotypic stability, all experiments were conducted using cells strictly between passages 5 and 10 after thawing.

### 2.4 Cell Viability and MTT Assay

Cell viability and the half‐maximal inhibitory concentration (IC_50_) were evaluated using the 3‐(4,5‐dimethylthiazol‐2‐yl)‐2,5‐diphenyltetrazolium bromide (MTT) assay. Briefly, MCF7 (6 × 10^3^ cells/well) and MDA‐MB‐231 (8 × 10^3^ cells/well) were seeded in 96‐well plates and incubated for 24 h. To determine the IC_50_ values, cells were treated with varying concentrations of abemaciclib (ranging from 1.25 to 40 μM) for 24, 48 and 72 h. Based on the cytotoxicity results, the 48‐h IC_50_ concentrations of 6.91 μM for MCF7 and 5.38 μM for MDA‐MB‐231 were selected for subsequent experiments. For the combination therapies, cells were treated with miR‐640 (50 nM), abemaciclib (IC_50_ dose) or their combination for 48 h. Following the treatments, 10 μL of MTT solution (5 mg/mL; Sigma‐Aldrich) was added to each well, and the plates were incubated at 37°C for 4 h. The supernatant was carefully discarded, and 100 μL of dimethyl sulfoxide (DMSO; Merck, Germany) was added to dissolve the formazan crystals. The plates were incubated in the dark for 30 min, and the absorbance was measured at 570 nm using a microplate reader (Multiskan FC, Thermo Fisher Scientific, USA). IC_50_ values and dose–response curves were generated using GraphPad Prism software.

### 2.5 miR‐640 Transfection

MCF7 and MDA‐MB‐231 cells were seeded in 6‐well plates 24 h prior to transfection and grown to approximately 70% confluence. miR‐640 mimics (5′‐ATGATCCAGGAACCTGCCTCT‐3′) were purchased from MedChemExpress (Cat. No. HY‐R01914) and transfected at a final concentration of 50 nM using Lipofectamine LipoFectMax 3000 Transfection Reagent (Cat. No. FP318) according to the manufacturer's instructions. During the transfection process, all groups (including the untreated control group) were cultured in FBS‐free and antibiotic‐free DMEM. After a 48‐h incubation at 37°C, transfection efficiency was validated via reverse transcription‐quantitative PCR (RT‐qPCR). A significant increase (*p* < 0.05) in miR‐640 expression levels post‐transfection was confirmed.

### 2.6 Annexin V Apoptosis Assay

Apoptosis was evaluated using the Annexin V/PI staining method. Cells were seeded into 6‐well plates (6 × 10^3^ for MCF7 and 8 × 10^3^ for MDA‐MB‐231 cells/well) and divided into four treatment groups: Control, Abemaciclib, miR‐640 and Combination. Following a 48‐h treatment period, cells were harvested, washed with 1× PBS, and resuspended in binding buffer. Cells were then stained with 2.5 μL Annexin V‐FITC and 2.5 μL propidium iodide (PI) using the Apoptosis Detection Kit II (BD Pharmingen, USA) according to the manufacturer's protocol. The stained cells were acquired and analysed using a BD Accuri C6 Plus flow cytometer.

### 2.7 Cell Cycle Assay

Cells were seeded into 6‐well plates as described above. Following 48 h of treatment with abemaciclib (IC_50_), miR‐640 (50 nM) or their combination, cells were harvested and processed using the Cell Cycle Analysis Kit (Ecotech CCA20) according to the manufacturer's instructions. For each group, approximately 2 × 10^4^ cells were acquired using a BD Accuri C6 flow cytometer. The distribution of cells in the G0/G1, S and G2/M phases was analysed and quantified using BD Accuri C6 Cflow Plus software (v1.0.264.15).

### 2.8 Wound Healing (Migration) Assay

MCF7 and MDA‐MB‐231 cells were cultured in 6‐well plates until they reached 100% confluence to form a monolayer. A linear scratch (wound) was created using a sterile 200 μL pipette tip. Cells were gently washed to remove debris and then exposed to the respective treatments (abemaciclib, miR‐640 or combination) in FBS‐free DMEM. The control group received only FBS‐free DMEM. The migration of cells into the wound area was photographed at specific predefined points at 0, 24 and 48 h. The relative wound closure area was quantified using ImageJ software (LOCI, University of Wisconsin).

### 2.9 Total RNA Isolation

Total RNA was extracted from the MCF7 and MDA‐MB‐231 cells using the RiboEx reagent (GeneAll Biotechnology, South Korea) according to the manufacturer's manual. RNA concentration and purity (A260/A280 absorbance ratio) were assessed using a NanoDrop 2000 spectrophotometer (Thermo Fisher Scientific, USA).

### 2.10 Quantitative Analysis of miR‐640 and mRNA Expression (RT‐qPCR)

For miR‐640 quantification, cDNA was synthesised using the miRNA All‐In‐One cDNA Synthesis Kit (Cat. No. G898). The RT‐qPCR was performed using a miR‐640 forward primer (ATGATCCAGGAACCTGCCTCT) and a universal reverse primer (Cat. No. MPH00000‐abm) with SYBR Green qPCR Master Mix (Cat. No. D026‐1). U6 snRNA (CTCGCTTCGGCAGCACA) served as the internal endogenous control. Additionally, for transfection time‐point optimisation, treatments were tested at 24 and 48 h, and the expression levels of two target genes (*ZFP91* and *MAP3K1*) were compared. The 48‐h treatment, which yielded the most profound downregulation, was selected for all assays.

For mRNA quantification, cDNA was synthesised using the OneScript Plus cDNA Synthesis Kit (Cat. No. G236). PCR amplification was conducted on a CFX Connect Real‐Time PCR Detection System (Bio‐Rad, USA). The thermocycling conditions were as follows: initial denaturation at 95°C for 3 min, followed by 40 cycles of 95°C for 15 s and 57°C for 1 min. Reactions were performed using the BlasTaq 2X qPCR MasterMix (Cat. No. G891) and the specific primers listed in Table 1. GAPDH was used as the reference gene. Relative expression levels of miRNAs and mRNAs were calculated using the 2^−ΔΔCt^ method. Data were analysed using Bio‐Rad CFX Maestro software (V1.1).

**TABLE 1:** Primers used for the quantification of gene expression by RT‐qPCR.

Gene	Forward primer (5′ → 3′)	Reverse primer (5′ → 3′)
*CDK4*	ATGGCTACCTCTCGATATGAGC	CATTGGGGACTCTCACACTCT
*CDK6*	CCAGGCAGGCTTTTCATTCA	AGGTCCTGGAAGTATGGGTG
*CDKN2B*	ACGGAGTCAACCGTTTCGGGAG	GGTCGGGTGAGAGTGGCAGG
*CDK9*	ACGGCCTCTACTACATCCACA	GCTGCGGGTCCACATCTCTGC
*E2F2*	CCTTGGAGGCTACTGACAGC	CCACAGGTAGTCGTCCTGGT
*SMAD2*	CCGACACACCGAGATCCTAAC	AGGAGGTGGCGTTTCTGGAAT
*WEE1*	GATGTGCGACAGACTCCTCAAG	CTGGCTTCCATGTCTTCACCAC
*WNT7B*	GAGCCAACATCATCTGCAACA	AACTGGTACTGGCACTCGTTGA
*ZFP91*	GCCATCGTGATACAGAGAACACC	CTAATGCCACCTGGAGACTGATG
*PDK1*	CTGTGATACGGATCAGAAACCG	TCCACCAAACAATAAAGAGTGCT
*MAP3K1*	CAACAACAACAACAACAGAG	TGAGGAGATGCAGAAGGT
*AURKA*	TGTGCCTTAACCTCCCTATTC	AACCTTGCCTCCAGATTATGA
*GAPDH*	GAGTCAACGGATTTGGTCGT	GACAAGCTTCCCGTTCTCAG

### 2.11 Statistical Analysis

All experiments, including functional assays and RT‐qPCR, were performed in at least three independent biological replicates—with each condition evaluated in technical triplicates—and data are presented as the mean ± standard deviation (SD). Statistical analyses and graphical representations were generated using GraphPad Prism software (version 9; GraphPad Software, USA). Differences between two independent groups were analysed using the unpaired Student's *t*‐test, whereas multi‐group comparisons (e.g., control vs. abemaciclib, miR‐640 and combination) were evaluated using a one‐way analysis of variance (ANOVA) followed by Tukey's post hoc multiple comparisons test. A *p* value of < 0.05 was considered statistically significant.

## 3 Results

### 3.1 In Silico Analysis Reveals Significant Downregulation of miR‐640 in Relapsed Breast Cancer

**FIGURE 1:**
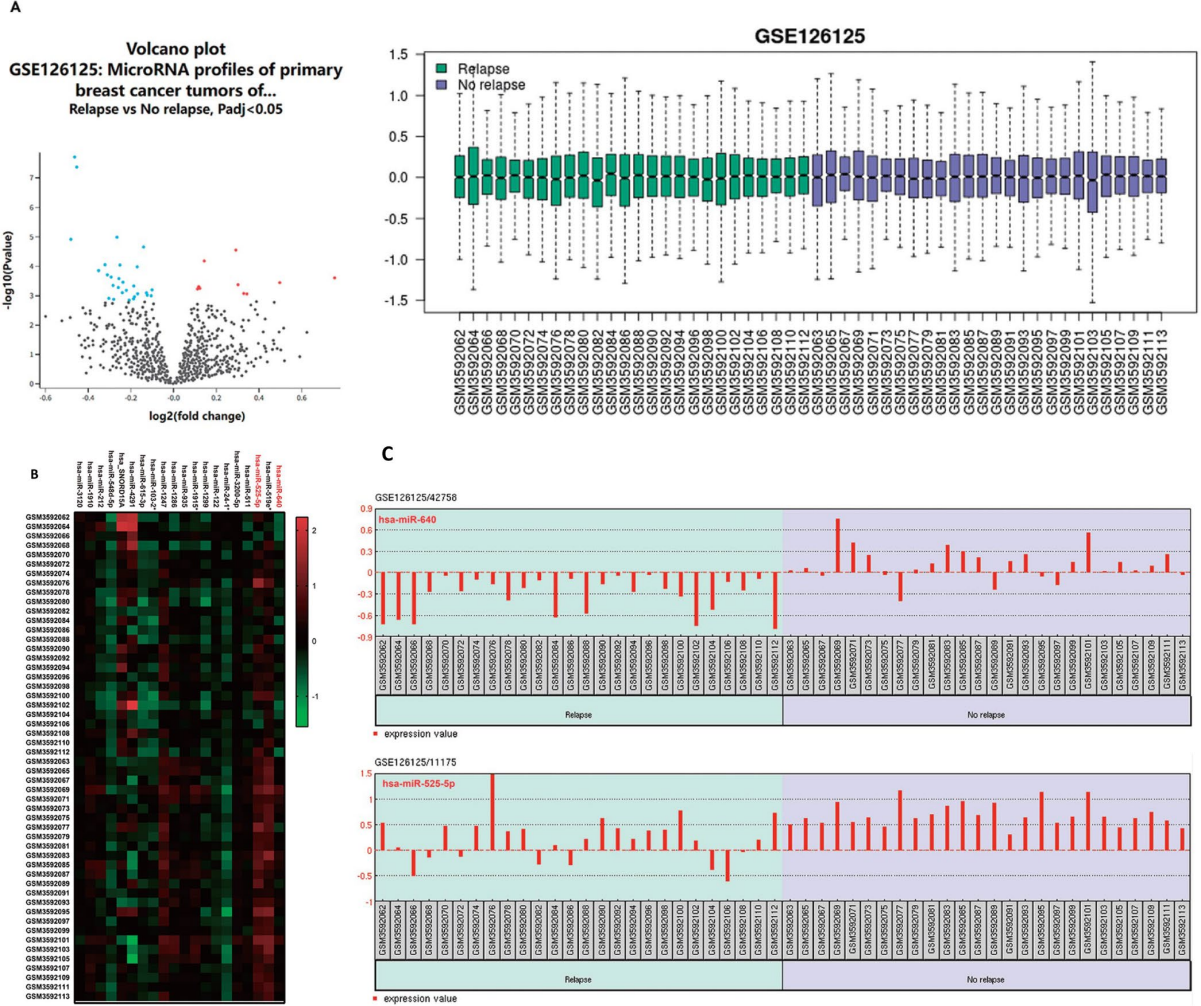
Analysis of microarray‐identified microRNAs associated with relapse of systematically untreated and lymph node negative patients with primary breast cancer retrieved from GEO accession GSE126125. (A) The data uploaded by Block I et al. (https://www.ncbi.nlm.nih.gov/geo/query/acc.cgi?acc=GSE126125) (GEO accession GSE126125, Platform GPL23960) was determined by GEO2R; Volcano plots presenting the differentially expressed microRNAs, and Box plot analysis indicating the comparably defined groups. (B) Heatmap of top 20 different microRNAs in tumours from patients with and without relapse. (C) Statistical analysis in GEO2R using the Bayes test showed differential expression of miR‐640 and miR‐525‐5p.

Analysis of the GSE126125 dataset comparing relapsed and non‐relapsed breast tumours demonstrated a significant differential expression of miR‐640. Volcano plot, box plot (Figure 1A) and heatmap (Figure 1B) analyses, along with Bayes test results (logFC = −0.4628, *p*adj < 0.001; Figure 1C), confirmed that miR‐640 was significantly downregulated in the relapse group compared to the non‐relapse group. These findings suggest a potential association between miR‐640 suppression and breast cancer recurrence.

### 3.2 In Silico Identification and Network Analysis of miR‐640 Target Genes Associated With CDKi Resistance

Through the cross‐referencing of miR‐640 target prediction databases with current literature on CDKi resistance mechanisms, a specific panel of resistance‐associated target genes was identified (Table 2).

**TABLE 2:** Genes involved in CDK4/6 inhibitor resistance and targeted by miR‐640.

Genes	Role in CDKi resistance	References
*CDK4*	CDK4 is a critical kinase within the Cyclin D‐CDK4/6‐RB signalling pathway. Various mechanisms such as gene amplification, mutations and epigenetic alterations activate the cyclin D‐CDK4/6‐RB pathway. The overexpression of *CDK4*, which has been identified in various cancers, may limit the efficacy of CDK4/6 inhibitors.	[19, 20, 21, 22]
*CDK6*	It plays an important role in the progression of the cell cycle from the G1 phase to the S phase. Increased expression of *CDK6* is associated with CDK4/6 inhibitor resistance and a reduced response to CDK4/6 inhibition by phospho‐RB (pRB). The function of CDK6 is primarily kinase‐dependent. However, CDK6 has a role in post‐transcriptional regulation independent of kinase activity; in the presence of STAT3 and cyclin D, it upregulates the post‐transcription of p16 in a kinase‐independent manner. Furthermore, CDK6, together with c‐Jun, upregulates vascular endothelial growth factor A (*VEGF‐A*), which is responsible for cancer progression and drug resistance due to its ability to induce angiogenesis.	[23, 24]
*CDKN2B*	*CDKN2B* encodes p15^INK4B^, a natural selective inhibitor of CDK4/6. Paradoxically, the overexpression of p15 (high *CDKN2B* levels) is strongly associated with intrinsic resistance to CDK4/6 inhibitors. This elevated expression indicates that tumour cells have bypassed the CDK4/6‐Rb pathway (e.g., via alternative CDK2 activation or Rb loss), rendering them independent of CDK4/6 activity. Consistent with this, high *CDKN2B* mRNA expression was validated as a prominent resistance marker to palbociclib in the NeoPalAna trial. Therefore, suppressing *CDKN2B* expression is a rational strategy to disrupt these bypass mechanisms and restore sensitivity to CDK inhibitors.	[25, 26, 27, 28]
*CDK9*	cdc2 (Cell Division Cycle 2‐CDK1) is a serine–threonine kinase associated with CDK1 and involved in numerous physiological processes. Unlike most cdc2‐like kinases, its activity is not regulated by the cell cycle. CDK9 primarily acts in processes distinct from cell cycle regulation, such as differentiation. It plays a role in cell cycle progression, growth, proliferation, differentiation and apoptosis. Increased CDK9 mRNA expression has been shown to be associated with resistance to CDK4/6 inhibitors. CDK9 inhibition reduces cMYC protein levels in MCF7 cells and also selectively inhibits the oestrogen‐independent growth of resistant cell lines, suggesting that CDK9 is a potential drug target in endocrine‐resistant breast cancer.	[29, 30, 31, 32]
*E2F2*	As a transcription factor, it plays an important role in regulating cell cycle progression from G1 to S phase. E2F amplification and cyclin E‐CDK2 complex formation are associated with resistance to CDK4/6 inhibitors.	[24, 26]
*SMAD2*	It triggers cell cycle arrest by inducing CDK inhibitors (p21^WAF1/CIP1^, p27^KIP1^ and p15^INK4B^) and inhibiting the expression of other cell cycle regulators such as c‐Myc and Cdc25A. However, SMAD2 activation also induces the expression of transcription factors for EMT, which may explain the tumour‐promoting effects of TGF‐β and activin in aggressive breast cancer. Furthermore, TGF‐β signalling leads to the phosphorylation of Smad2 and Smad3, which then form a complex with Smad4, leading to EMT through the activation of transcription factors involved in EMT.	[33, 34]
*WEE1*	It is a serine/threonine kinase that plays a role in ensuring accurate DNA replication, regulates the G2/M checkpoint, and phosphorylates and inhibits CDK1 and CDK2. Although the role of WEE1 in inducing resistance to CDK4/6 inhibitors is unknown, inhibition of WEE1 has been shown to increase sensitivity to CDK4/6 inhibitors in resistant cells.	[24, 35]
*WNT7B*	As a ligand belonging to the Wnt family, it activates the Wnt/β‐catenin signalling pathway. The Wnt/β‐catenin pathway promotes tumour growth, particularly in cancer cells, by increasing the expression of cell cycle regulators (e.g., Cyclin D1). Studies exist on the contribution of WNT7B to treatment resistance in cancers in general. Although there is no direct study on the mechanism of creating genetic resistance specific to CDK inhibitors, it has been reported that it plays an important role in treatment resistance in cancers, particularly in the resistance mechanism against immune checkpoint inhibitors.	[36, 37, 38]
*ZFP91*	E3 ubiquitin ligase activity supports cell survival by activating the WNT, NF‐kB and MAP kinase signalling pathways and plays an important role in resistance to immunomodulatory drugs. These signalling pathways may also affect the expression of genes that regulate the cell cycle and play a role in resistance to CDK4/6 inhibitors. Furthermore, ZFP91's ubiquitin ligase activity and autophagy regulatory functions may also be effective in the adaptation of cells to stress conditions.	[39]
*PDK1*	It is a kinase that targets the PI3K signal downstream and AKT upstream, and is an important regulator of ribociclib sensitivity in ER+ BC cells. PDK1 phosphorylation is frequently increased and is significantly associated with BC invasiveness.	[40, 41]
*MAP3K1*	Activation of the MAPK signalling pathway and many of its downstream effectors, such as RAS–RAF–MEK–ERK, has been observed in CDK4/6i‐resistant models. Indeed, in BC cell lines with acquired resistance to CDK4/6i, the use of MEK inhibitors in combination with CDK4/6i is effective in inhibiting cell proliferation, supporting a possible role for the MAPK pathway in acquired resistance to CDK4/6i.	[42]
*AURKA*	*AURKA* plays a key role in controlling chromosome assembly and segregation during mitosis. It shows increased expression in tumours resistant to CDK4/6 inhibitors. *AURKA* amplification was found in tumour biopsies taken from patients resistant to CDK4/6i treatment, while no changes were detected in samples taken from patients sensitive to CDK4/6i.	[42]

**FIGURE 2:**
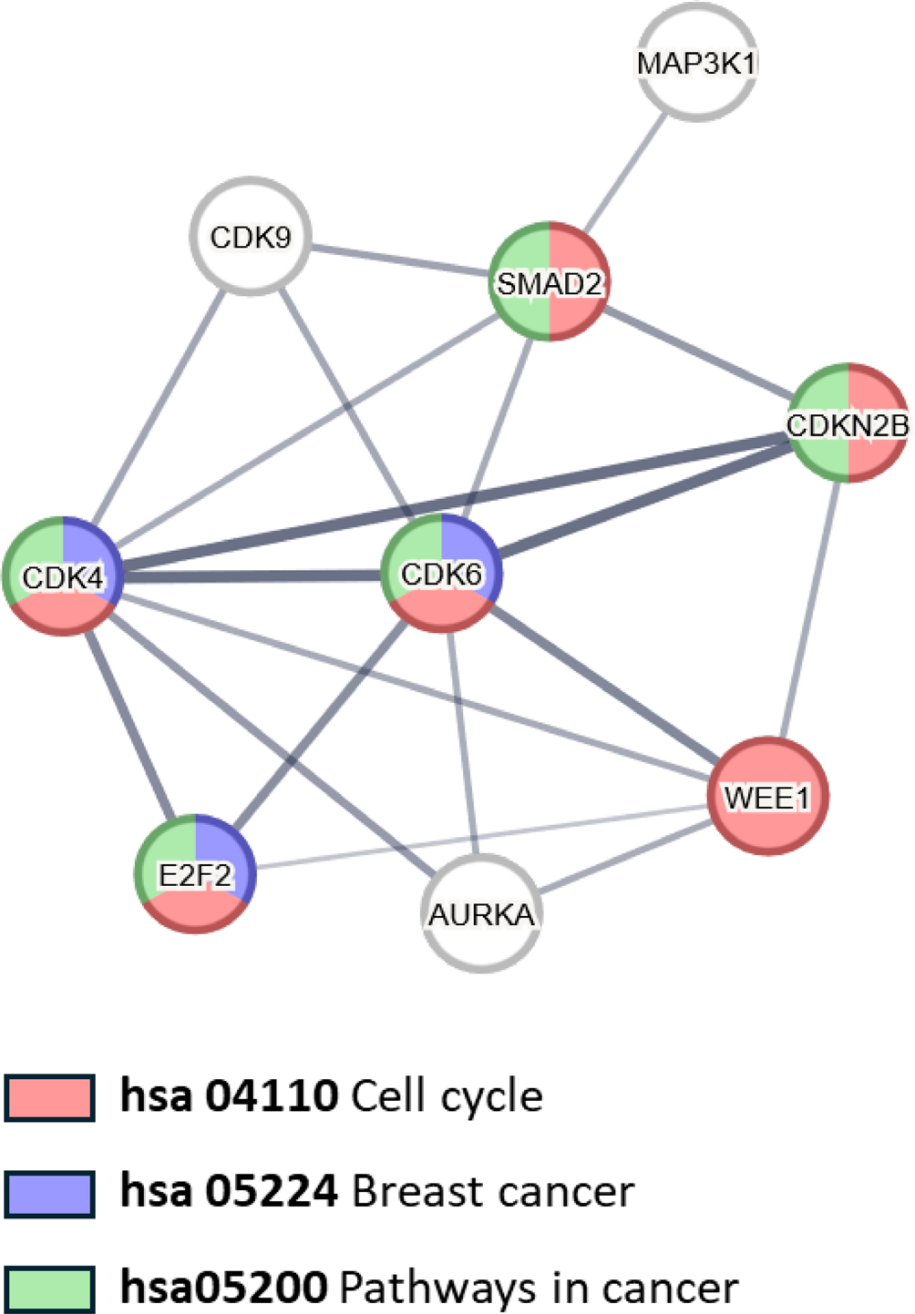
Protein–protein interaction (PPI) network and KEGG pathway enrichment analysis of the selected CDKi resistance‐associated target genes. The network was constructed using the STRING database (version 12.0) with a medium confidence interaction score (0.400). Disconnected nodes were hidden to focus strictly on the highly interactive core module. The nodes represent the target genes, and the line thickness indicates the strength of data support for the interactions. The core genes are significantly enriched in key oncogenic KEGG pathways, denoted by the pie chart colour codes: red for Cell cycle (hsa04110), blue for Breast cancer (hsa05224) and green for Pathways in cancer (hsa05200).

Before assessing the post‐transcriptional regulatory capacity of miR‐640 in vitro, the functional interconnectivity of the selected CDKi resistance‐associated genes was validated using STRING PPI network analysis. At a medium confidence score (0.400), 9 of the targeted genes formed a highly cohesive core interaction network. KEGG pathway enrichment analysis revealed that this core module is significantly clustered within critical oncogenic networks, predominantly ‘Cell cycle’ (hsa04110), ‘Breast cancer’ (hsa05224) and ‘Pathways in cancer’ (hsa05200) (Figure 2). Notably, key cell cycle regulators such as CDK4, CDK6 and E2F2 emerged as central hubs, intersecting strongly across these enriched pathways, underscoring their pivotal role in the resistance machinery.

### 3.3 Quantitative Validation of miR‐640 Transfection

**FIGURE 3:**
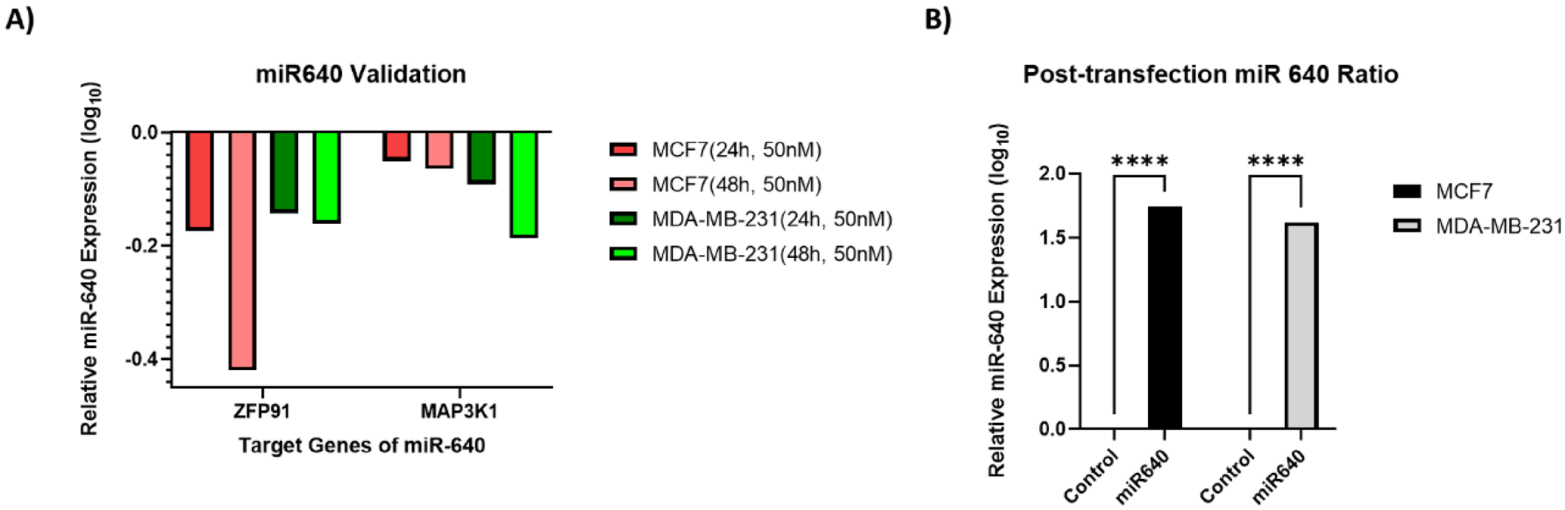
(A) Time‐dependent silencing efficacy of miR‐640 on target genes. (B) Relative expression levels of miR‐640 following transfection in MCF7 and MDA‐MB‐231 cell lines (control: untreated; miR‐640: miRNA transfection alone). Data are expressed as mean ± SD (****p < 0.0001).

To ensure the reliability of the subsequent functional assays, the efficiency of miR‐640 cellular uptake was validated. Following the transfection of MCF7 and MDA‐MB‐231 cells with 50 nM miR‐640 mimics, RT‐qPCR analysis confirmed a dramatic and statistically significant upregulation of miR‐640 expression in both MCF7 (~50‐fold) and MDA‐MB‐231 (~42‐fold) cell lines (represented as ~1.7 and ~1.6 on the Log10 scale, respectively) compared to the untreated controls (*p* < 0.0001) (Figure 3B). Additionally, time‐dependent suppression analysis of target genes (*ZFP91* and *MAP3K1*) confirmed that a 48‐h transfection period yielded the optimal gene silencing effect (Figure 3A), establishing this timeframe for all subsequent experiments.

### 3.4 miR‐640 Sensitises Breast Cancer Cells to Abemaciclib‐Induced Cytotoxicity

**FIGURE 4:**
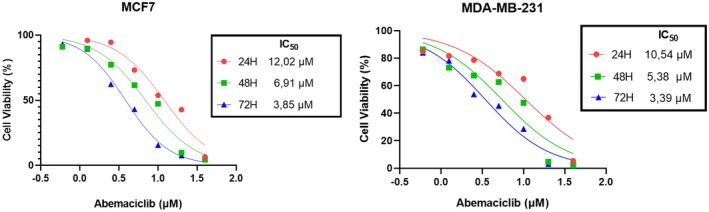
Time‐dependent cytotoxicity of abemaciclib in breast cancer cells. IC_50_ values of MCF7 and MDA‐MB‐231 cell lines at 24, 48 and 72 h post‐treatment, calculated via non‐linear regression analysis (*R* ^2^ > 0.90).

To determine the baseline anti‐proliferative effect of abemaciclib, cells were treated with varying concentrations (1.25–40 μM) for 24, 48 and 72 h. Dose–response curves indicated a time‐ and dose‐dependent inhibition of cell viability (Figure 4). The 48‐h IC_50_ values were calculated as 6.91 μM for the MCF7 line and 5.38 μM for the MDA‐MB‐231 line.

**FIGURE 5:**
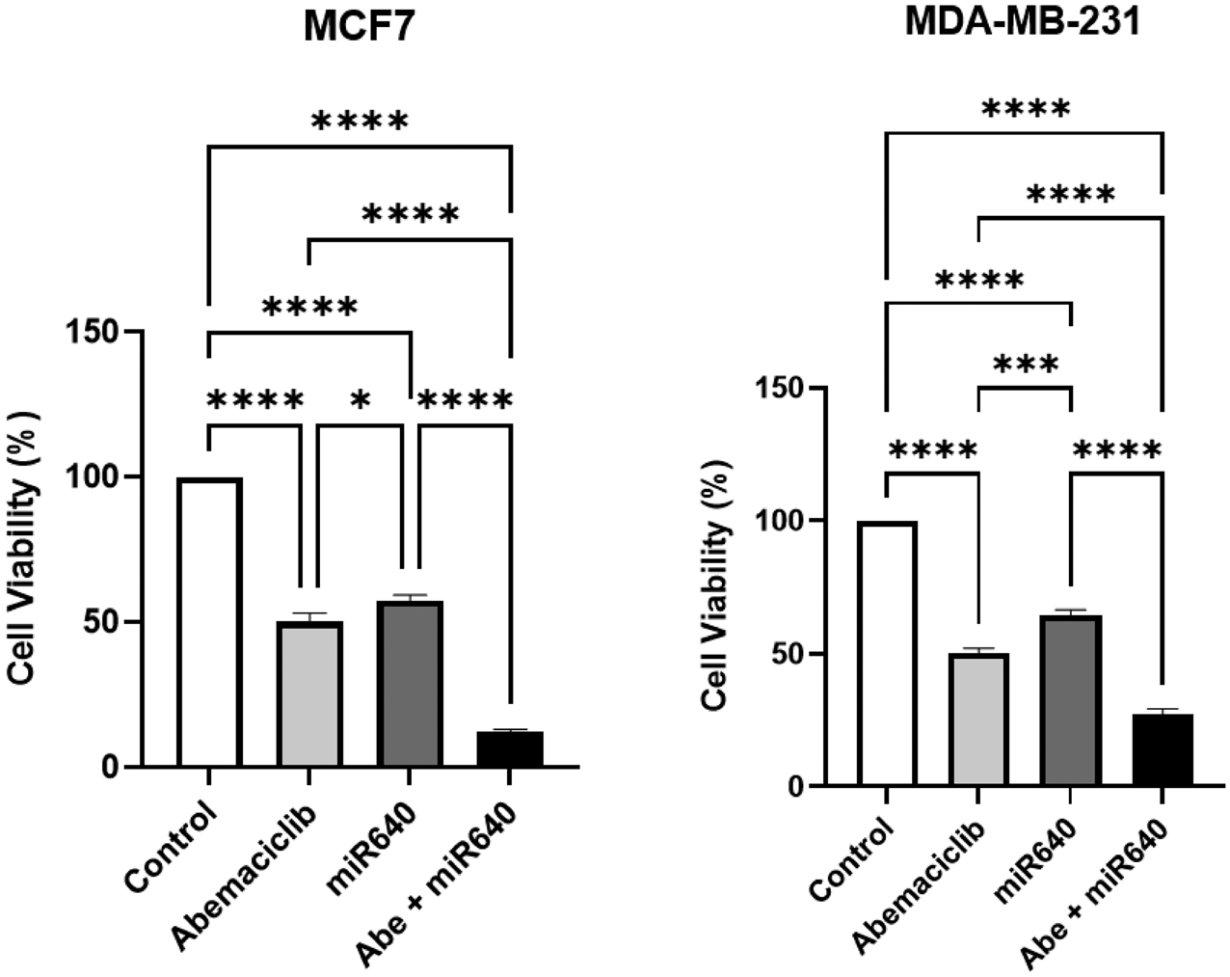
Combinatorial effect of miR‐640 and abemaciclib on cell viability. Relative viability percentages of MCF7 and MDA‐MB‐231 cell lines following treatment with abemaciclib (IC50 dose) alone, miR‐640 transfection alone or their combination, compared to untreated controls. Data are expressed as mean ± SD (*p < 0.05, ***p < 0.001, ****p < 0.0001).

Upon assessing the combinatorial therapeutic effect, the administration of 50 nM miR‐640 alone significantly reduced cell viability by approximately 40% in MCF7 and 30% in MDA‐MB‐231 cells (*p* < 0.05) compared to controls. Remarkably, the combination of abemaciclib (at IC_50_) and miR‐640 induced a pronounced additive suppression, markedly reducing cell viability to approximately 20% in MCF7 and 35% in MDA‐MB‐231 cells (*p* < 0.0001). This combinatorial inhibition was significantly more potent than the effect of either agent alone (Figure 5).

### 3.5 Combination of miR‐640 and Abemaciclib Additively Induces Apoptosis

**FIGURE 6:**
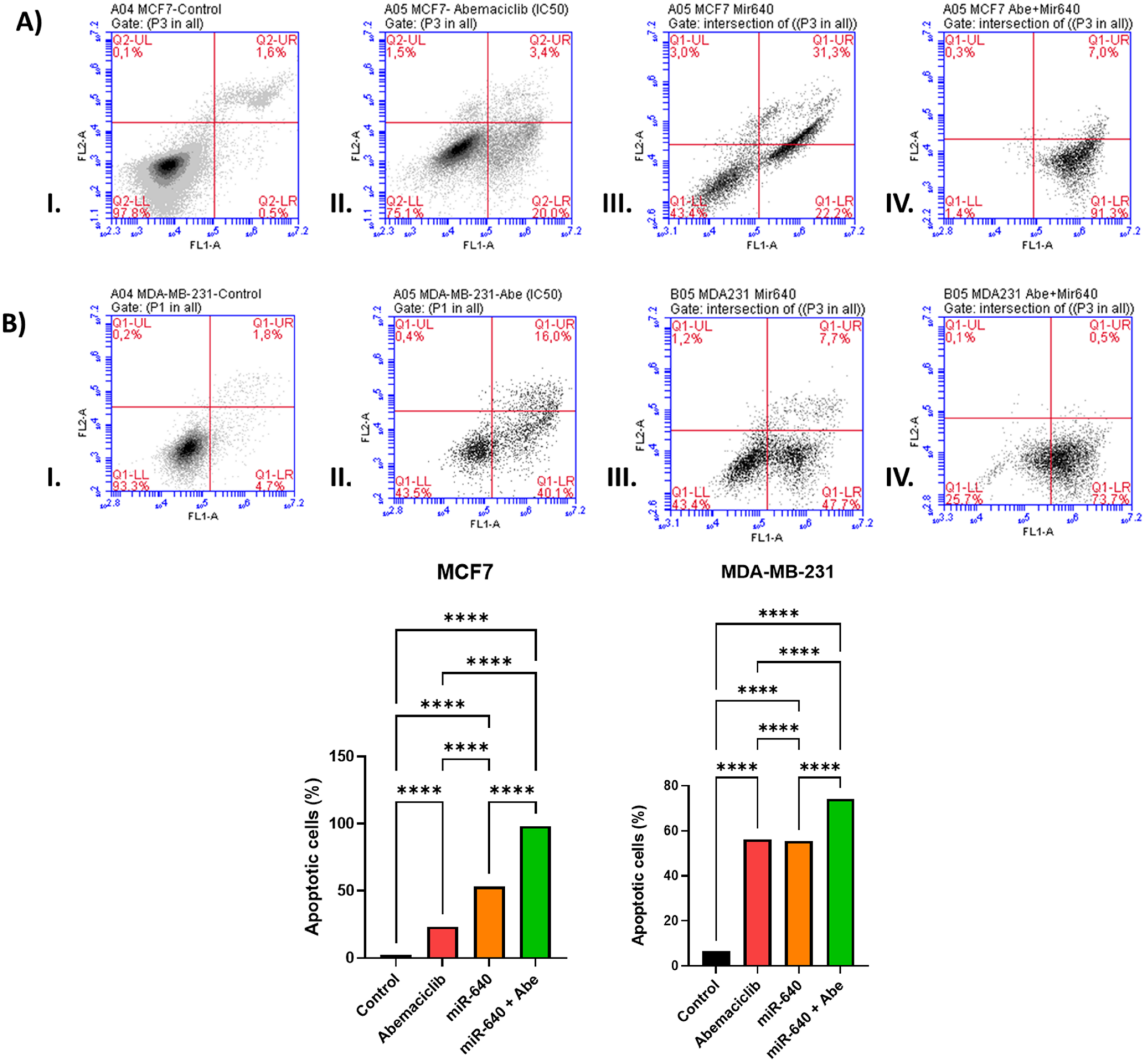
Combinatorial effect of miR‐640 and abemaciclib on cellular apoptosis. Comparative analysis of apoptotic effects in MCF7 and MDA‐MB‐231 cell lines following indicated treatments. Experimental groups include untreated control, miR‐640 transfection alone (50 nM), abemaciclib treatment alone (IC50 dose), and their combination (abemaciclib + miR‐640). Data are expressed as mean ± SD (****p < 0.0001).

Annexin V/PI flow cytometry analysis was performed to evaluate the apoptotic impact of the treatments. In both MCF7 (Figure 6A) and MDA‐MB‐231 (Figure 6B) cell lines, single‐agent treatments (abemaciclib or miR‐640 alone) significantly induced apoptosis compared to the control groups. Crucially, the concurrent administration of abemaciclib and miR‐640 elicited a robust apoptotic response, demonstrating a significant additive apoptotic effect compared to individual monotherapies.

### 3.6 miR‐640 Alters Cell Cycle Dynamics and Induces Phase‐Specific Arrests

**FIGURE 7:**
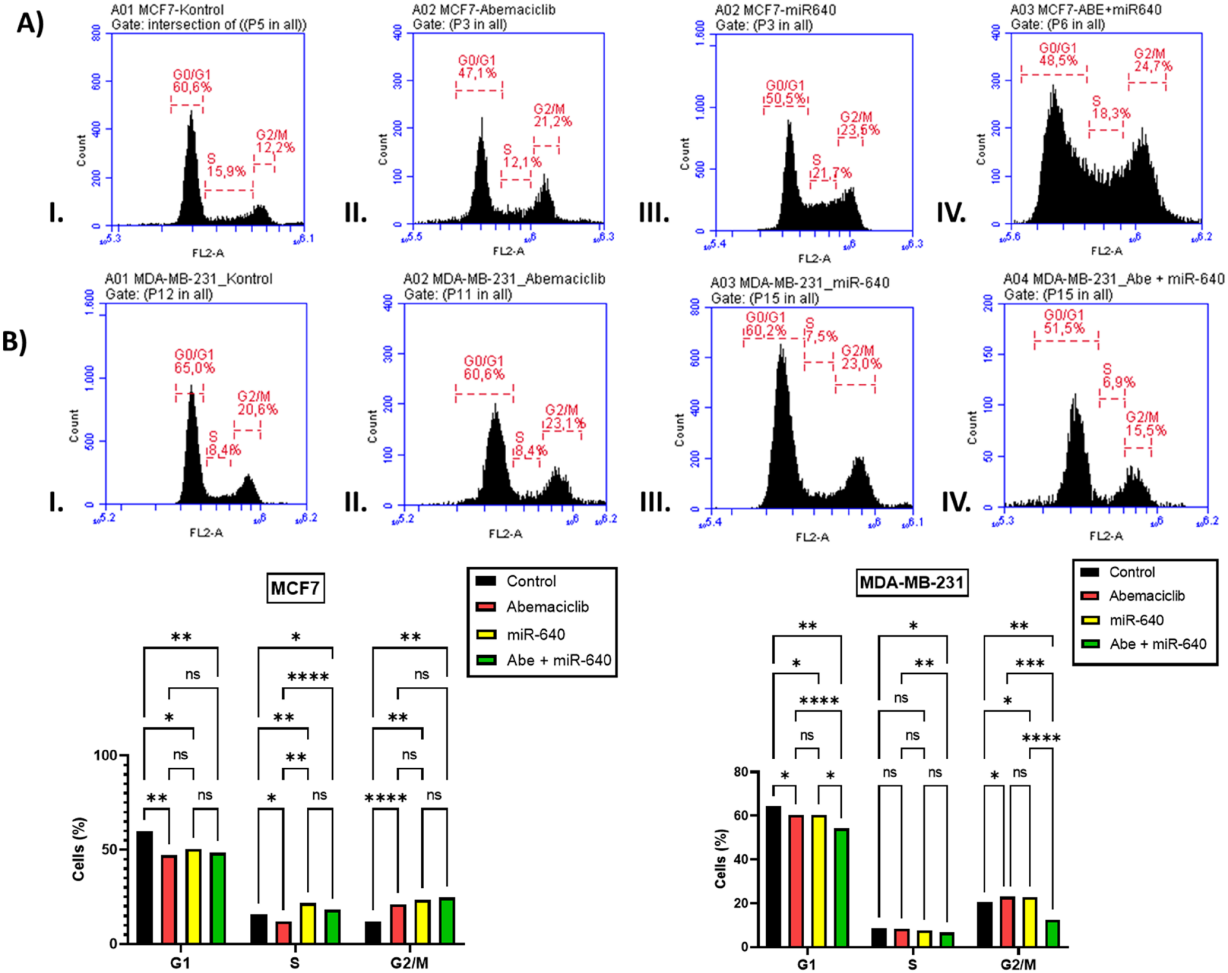
Combinatorial effect of miR‐640 and abemaciclib on cell cycle distribution. Representative flow cytometry histograms and quantitative analysis of cell cycle phases (G0/G1, S and G2/M) in (A) MCF7 and (B) MDA‐MB‐231 cell lines following indicated treatments. Experimental groups include untreated control, abemaciclib treatment alone, miR‐640 transfection alone, and their combination. Data are expressed as mean ± SD (*p < 0.05, **p < 0.01, ***p < 0.001, ****p < 0.0001, ns: not significant).

Flow cytometry was utilised to determine the distribution of cells across cell cycle phases (Figure 7). In MCF7 cells (Figure 7A), abemaciclib monotherapy decreased the G0/G1 population (47.1%) and increased the G2/M population (21.2%) compared to controls. miR‐640 monotherapy increased both S (21.7%) and G2/M (23.5%) phases. The combination treatment resulted in a pronounced shift, retaining cells primarily in the S and G2/M phases.

Conversely, in MDA‐MB‐231 cells (Figure 7B), while abemaciclib monotherapy caused a slight accumulation in the G2/M phase (23.1%), miR‐640 monotherapy predominantly retained cells in the G0/G1 phase (60.3%). Interestingly, the combination treatment significantly reduced both S (6.9%) and G2/M (15.5%) phase populations, arresting the majority of the cells (51.5%) in the G0/G1 phase.

### 3.7 miR‐640 and Abemaciclib Combination Profoundly Inhibits Cell Migration

**FIGURE 8:**
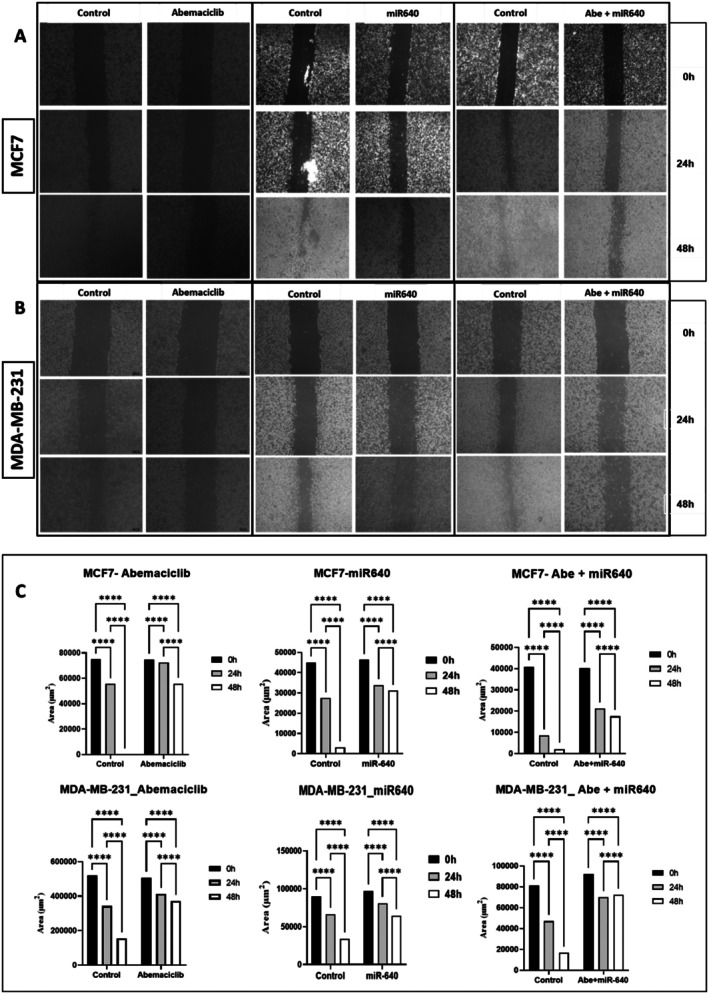
Combinatorial effect of miR‐640 and abemaciclib on cell migration. Representative phase‐contrast microscopy images showing the in vitro scratch wound healing process over 48 h in (A) MCF7 and (B) MDA‐MB‐231 breast cancer cells following treatment with abemaciclib (IC50 dose) alone, miR‐640 transfection alone or their combination, compared to untreated controls. (C) Quantitative analysis of the percentage of wound closure over time for both cell lines. Data are expressed as mean ± SD. Asterisks indicate statistical significance (****p < 0.0001).

The anti‐metastatic potential of the treatments was evaluated using a wound‐healing assay over 48 h (Figure 8A,B). Quantitative analysis of the wound closure area revealed that both abemaciclib and miR‐640 monotherapies significantly delayed cellular migration compared to the rapid wound closure observed in control groups at 24 and 48 h. Most notably, the combination of abemaciclib and miR‐640 markedly reduced cell migration in both MCF7 and MDA‐MB‐231 cell lines, maintaining a significantly larger open wound area (*p* < 0.0001) (Figure 8C).

### 3.8 miR‐640 Post‐Transcriptionally Downregulates CDKi Resistance‐Associated Gene Networks

**FIGURE 9:**
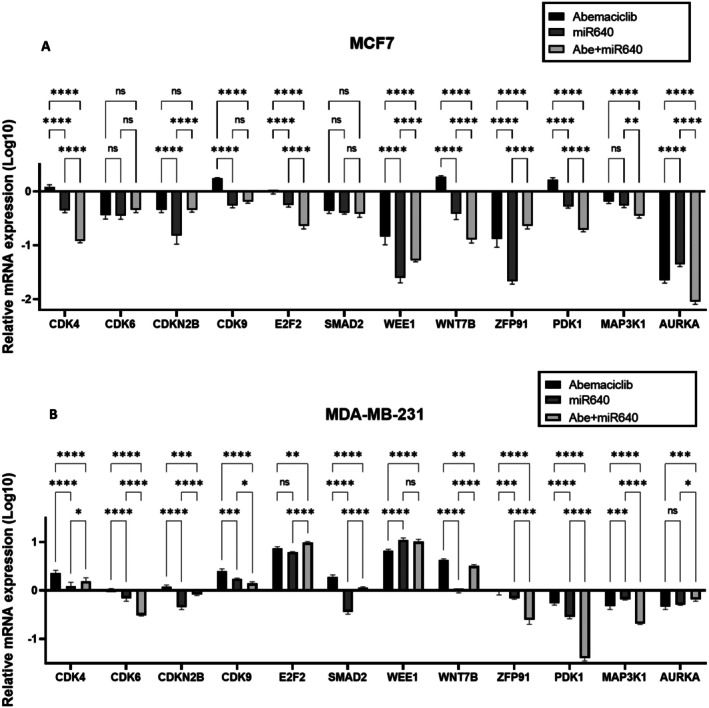
Combinatorial effect of miR‐640 and abemaciclib on target gene expression. Comparative analysis of relative mRNA expression levels in MCF7 (A) and MDA‐MB‐231 (B) cell lines following treatment with abemaciclib alone, miR‐640 transfection alone or their combination. Gene expression fold changes are normalised to untreated controls and presented on a logarithmic scale (Log10). Data are expressed as mean ± SD (*p < 0.05, **p < 0.01, ***p < 0.001, ****p < 0.0001, ns: not significant).

To elucidate the molecular mechanisms underlying the observed functional phenotypes, the expression levels of 12 selected CDKi resistance‐associated genes were quantified via RT‐qPCR (Figure 9). In the MCF7 cell line, miR‐640 transfection induced a robust and significant downregulation across the entire targeted gene panel, with the most profound suppressions observed in WEE1, ZFP91 and *AURKA*. In the MDA‐MB‐231 cell line, miR‐640 caused a strong downregulation of PDK1, alongside moderate but significant suppressions of *CDK6, CDKN2B, SMAD2, ZFP91, MAP3K1* and *AURKA*. The combination of abemaciclib and miR‐640 consistently maintained or further enhanced these post‐transcriptional suppressions in both cell lines, confirming that miR‐640 effectively disrupts the resistance‐associated gene network at the mRNA level.

## 4 Discussion

The primary objective of this study was to evaluate the potential of overcoming abemaciclib resistance in breast cancer treatment and to investigate the therapeutic effects of miR‐640 on cell proliferation, apoptosis and migration by regulating resistance‐associated genes. Herein, we provide the first evidence that the comprehensive target gene network driving CDKi resistance is profoundly suppressed by miR‐640, culminating in significant migration inhibition and apoptosis induction at the cellular level. While current literature highlights miR‐640 as a tumour suppressor and angiogenesis inhibitor in various malignancies, including hepatocellular carcinoma, ovarian cancer and breast cancer [15, 16, 17, 18], its specific regulatory role in CDKi resistance had not been previously elucidated. Our study addresses this critical therapeutic gap, offering a novel and mechanistic contribution to the oncology literature.

The foundational rationale of our target selection was based on the in silico observation from the GSE126125 dataset, which revealed a significant downregulation of miR‐640 in relapsed breast cancer patients. Crucially, the clinical validity of this specific transcriptomic profile has been recently and robustly confirmed in an independent clinical cohort by Tang et al. [43]. In their comprehensive validation study, quantitative RT‐qPCR analyses of fresh‐frozen tumour biopsies from 180 primary breast cancer patients definitively demonstrated that miR‐640 expression is significantly reduced in patients who develop clinical recurrence. This external clinical validation, utilising actual patient tissues, not only compensates for the lack of in vivo models in the current study but also strongly reinforces the translational relevance of our in silico findings, firmly justifying the investigation of miR‐640 as a therapeutic target against abemaciclib resistance.

Consistent with previous reports utilising miRNA mimics [44, 45], the successful cellular uptake of miR‐640 in our model effectively resensitised breast cancer cells to abemaciclib (Figure 5). The significant additive apoptotic response observed upon combining miR‐640 with abemaciclib is in complete concordance with existing literature reporting the individual apoptotic impacts of these agents [46, 47, 48, 49, 50].

Cell cycle dynamics revealed notable cell‐type‐specific responses. In hormone‐responsive MCF7 cells, miR‐640 monotherapy exerted a strong independent suppressive effect, causing a distinct arrest in the G0/G1 phase. While abemaciclib monotherapy induced a cycle response parallel to the cellular heterogeneity and partial resistance development reported in the literature [26, 51], the combination of these two agents profoundly altered cycle dynamics, arresting MCF7 cells primarily in the S and G2/M phases. The marked inability of these cells to resolve such late‐phase arrests correlates directly with the significant apoptotic cell death observed in our Annexin V assays, as permanently arrested cells are ultimately directed toward programmed cell death. Conversely, in the triple‐negative breast cancer (TNBC) MDA‐MB‐231 cell line, miR‐640 predominantly retained cells in the G0/G1 phase. Interestingly, the combination therapy bypassed the classical G0/G1 arrest typically seen with abemaciclib and elicited a distinct cycle response, significantly reducing both S and G2/M phase populations. This strongly aligns with the innate/partial resistance and alternative cycling mechanisms typically exhibited by TNBC cells against CDK4/6 inhibitors [35, 52, 53].

In our wound‐healing model, miR‐640 monotherapy significantly delayed cell motility, establishing a substantial anti‐metastatic barrier on its own. However, the significant reduction of cell migration by the combination treatment strongly suggests that this additive interaction enhances the therapeutic potential. This dramatic inhibition of migration is robustly supported by similar miRNA/abemaciclib interventions reported for MDA‐MB‐231 [49, 54, 55] and MCF7 [56, 57] cell lines in the literature.

One of the most striking findings of our study is the robust post‐transcriptional suppression of the CDKi resistance‐driving gene network by miR‐640, exhibiting cell‐type‐specific dynamics. This widespread suppressive effect can be evaluated along two main mechanistic axes:

*1. Suppression of cell cycle and mitotic regulators*: The foundation of resistance to CDK4/6 inhibitors typically involves the overexpression of CDK4, CDK6 and E2F family members, which bypasses abemaciclib‐induced suppression [19, 21, 24, 35, 42, 58]. The simultaneous post‐transcriptional downregulation of these central hubs (CDK4, CDK6 and E2F2) by miR‐640 effectively dismantles this primary escape mechanism, providing a molecular basis for the pronounced anti‐proliferative effects. Interestingly, the cell‐type‐specific variations in this suppression profile suggest that while cycle inhibition is paramount in hormone‐responsive phenotypes, the therapeutic efficacy in TNBC models relies more heavily on disrupting alternative signalling cascades. Furthermore, the targeted suppression of CDKN2B (p15)—a recognised palbociclib resistance marker [59]—and the G2/M checkpoint regulator WEE1 [42, 60] critically reinforces this cycle blockade and supports the additive cytotoxicity observed in combination therapies. Additionally, modulating AURKA‐driven survival pathways and CDK9 expression further consolidates this multi‐nodal interference, exploiting the vulnerability of resistant cells to mitotic disruption [61, 62, 63, 64]. The robustness of this multi‐nodal blockade is further supported by our PPI network analysis. The established network demonstrates a highly interactive core primarily clustered within the ‘Cell cycle’ and ‘Breast cancer’ pathways, with CDK4, CDK6 and E2F2 acting as heavily interconnected central hubs. The simultaneous post‐transcriptional downregulation of this physical core by miR‐640 effectively dismantles the cooperative cell cycle signalling required for abemaciclib resistance.

*2. Suppression of alternative survival and metastasis pathways*: Abemaciclib resistance is frequently characterised by the compensatory hyperactivation of bypass survival routes, including the PI3K/AKT and MAPK pathways [23, 65]. Given that the PI3K/PDK1 pathway supports survival in ribociclib‐resistant models [24, 40, 41, 66], our observation that miR‐640 downregulates PDK1 indicates an effective suppression of this alternative survival route. In parallel, the concurrent repression of MAP3K1 underscores the capacity of miR‐640 to simultaneously dismantle multiple kinase‐driven resistance mechanisms. Beyond proliferation and survival, therapeutic resistance is intricately linked to metastatic progression and the tumour microenvironment [33, 37, 67, 68, 69, 70]. The coordinated downregulation of genes governing angiogenesis and Epithelial‐Mesenchymal Transition (EMT), namely WNT7B, ZFP91 and SMAD2, mechanistically explains the robust anti‐metastatic phenotype observed in our functional assays. This suggests that miR‐640 not only restricts intrinsic survival pathways but also broadly modulates the microenvironmental factors driving aggressive cancer progression.

Furthermore, while genes such as PDK1, ZFP91 and WNT7B did not show direct high‐confidence physical interactions with this central cell cycle core in the STRING analysis, their profound suppression by miR‐640 in vitro is biologically critical. It indicates that miR‐640 not only halts direct cell cycle progression but concurrently abrogates parallel bypass survival and metastatic mechanisms (such as PI3K/AKT and Wnt signalling). This dual‐action regulatory capacity explains the significant additive apoptotic response and marked migration inhibition observed in our combination therapies.

While the present study comprehensively maps the miR‐640‐mediated post‐transcriptional repression of this resistance network at the mRNA level, we acknowledge that miRNA‐induced gene silencing ultimately culminates in translational repression. Although protein‐level validations (e.g., Western blot analysis) of these specific multi‐gene targets fall outside the current scope, the dramatic phenotypic responses observed in our in vitro functional assays—namely, the marked reduction of cell migration, profound cell cycle arrests and substantial apoptotic induction—strongly corroborate the functional translation of this multi‐target silencing. The robust correlation between our transcriptomic data and these profound phenotypic shifts provides a compelling mechanistic foundation that warrants further protein‐level and in vivo investigations.

## 5 Conclusion

In recent years, miRNA replacement therapies have gained significant momentum [71, 72], and targeting multiple components within a signalling network using a single miRNA holds the potential to provide sustainable therapeutic benefits. To our knowledge, this study provides initial evidence that miR‐640 represents a promising and innovative therapeutic agent capable of overcoming CDK4/6 inhibitor resistance through comprehensive multi‐gene targeting.

To strengthen the clinical translational potential of these pioneering findings, it is of critical importance to evaluate this approach across broader cell line panels representing diverse breast cancer subtypes and to advance to in vivo (xenograft) animal models. Furthermore, a detailed elucidation of the specific molecular targets and downstream signalling networks of miR‐640 utilising high‐throughput next‐generation sequencing (NGS)‐based RNA‐Seq analysis is highly recommended. These forthcoming steps will enable a comprehensive understanding of the molecular mechanisms underlying miR‐640 activity and clarify its prognostic and predictive value, ultimately establishing a robust foundation for the integration of these findings into personalised breast cancer treatment strategies.

## Author Contributions

**Cigir Biray Avci:** writing – review and editing, supervision. **Ismail Mert Alkac:** writing – original draft, methodology, validation, visualization, writing – review and editing, formal analysis.

## Funding

This research was supported by the Ege University Scientific Research Projects Coordination Office (Grant No. 30785) and the The Scientific and Technological Research Council of Türkiye (TÜBİTAK) (Grant No. 125Z093).

## Ethics Statement

The authors have nothing to report.

## Consent

The authors have nothing to report.

## Conflicts of Interest

The authors declare no conflicts of interest.

## Data Availability

All data generated or analysed during this study are included in this published article, and the raw datasets are available from the corresponding author upon reasonable request.
